# Linking systemic angiogenic markers to synovial vascularization in rheumatoid arthritis

**DOI:** 10.1371/journal.pone.0203607

**Published:** 2018-09-06

**Authors:** Agathe Leblond, Sonia Pezet, Anne Priscille Trouvin, Muriel Elhai, Virginie Gonzalez, Yannick Allanore, Jérôme Avouac

**Affiliations:** 1 Université Paris Descartes, Sorbonne Paris Cité, INSERM U1016 and CNRS UMR8104, Institut Cochin, Paris, France; 2 Université Paris Descartes, Sorbonne Paris Cité, Service de Rhumatologie A, Hôpital Cochin, Paris, France; University of Texas Southwestern Medical Center, UNITED STATES

## Abstract

**Background:**

Neoangiogenesis is a crucial event to promote the development of the hyperplasic proliferative pathologic synovium in Rheumatoid arthritis (RA). Ultrasound (US) is sensitive for detection of power Doppler (PD) vascularization.

**Objective:**

To explore the associations between a set of complementary circulating angiogenic markers and a comprehensive US assessment in patients with RA.

**Patients and methods:**

Serum levels of eight angiogenic markers were measured by quantitative ELISAs in a total of 125 patients with RA, who were all systematically assessed in parallel by PDUS, performed on 32 joints.

**Results:**

Serum levels of soluble Vascular Cell Adhesion Molecule-1 (sVCAM-1) and Tie-2 were more likely to be increased in patients with synovial hyperemia detected on at least one joint (Power Doppler grade ≥1). sVCAM-1, Tie-2 and Angiostatin concentrations gradually increased together with the grade of the semiquantitative PDUS scale and concentrations of these three markers were markedly increased in patients with moderate to marked hyperemia (Power Doppler grade 2 and 3). Levels of sVCAM-1, Tie-2, and Angiostatin correlated with a global arthritis sum score, defined by the sum of the semiquantitative PDUS scores for all joints examined. Levels of Tie-2 and Placenta Growth Factor (PlGF) were associated with PDUS features indicating residual disease activity.

**Conclusion:**

Our results support the relevance of measuring serum levels of vascular markers to evaluate the intensity and extent of synovial vascularization. Angiogenic markers, and particularly Tie-2, could be a valuable surrogate of active synovitis and their place in relation to PDUS in clinical practice deserve further investigation.

## Introduction

Rheumatoid arthritis (RA) is the most common cause of chronic inflammatory arthritis with a prevalence ranging from 0.5% to 1% of the adult population worldwide [1, 2]. It is an autoimmune disease with a complex pathogenesis implicating innate and adaptive immunity together with angiogenesis [3].

The synovium is the primary site of RA-related inflammatory process, with infiltration of blood-derived inflammatory cells at the interface between cartilage and bone. One of the most noticeable signs of synovitis is the amount of synovial vascularization related to angiogenesis and vasculogenesis, which are critical for synovial proliferation and invasiveness. This invasive and destructive front promotes the development of bone and cartilage destruction.

Formation of new vessels consists of several complementary processes including activation, proliferation and migration of endothelial cells. This phenomenon is mediated by the differential regulation of angiogenic mediators and inhibitors [4–6].

Neoangiogenesis leads, together with inflammation-induced vasodilation of preexistent blood vessels, to increased blood flow in affected joints. Previous studies showed the considerable ability of highly sensitive power Doppler Ultrasound (PDUS) to improve the scoring of synovitis by detecting extended synovial vasculature [7, 8]. In addition, persistent synovial vascularity, assessed by power Doppler ultrasound (PDUS), has been linked to increased risk of disease flares and structural joint damages [9, 10].

Only scarce data are currently available regarding correlations between systemic angiogenic activity, measured by angiogenic factors in the serum, and the amount of local synovial vascularization measured by Doppler ultrasound [11]. Previous studies were characterized by limited sample size and assessed a restricted set of angiogenic markers, focusing mainly on VEGF. Moreover, the relationship between residual synovial vascularization and circulating angiogenic marker levels in patients with low disease activity (LDA) has not been explored in detail so far.

Acute phase reactants (ESR and CRP) are largely used in clinical practice but may not continually reflect the persistence of synovitis. Thus, novel biomarkers that exhibit higher associations with inflammation and angiogenesis in RA patients are warranted. The objective of the present study was to seek for associations between synovial vascularity assessed by PDUS and a panel of 8 serum vascular markers, reflecting different angiogenic processes, such as endothelial cell activation, proliferation, survival, growth and migration, as well as vessel maturation and stabilization.

## Patients and methods

### Study design

Cross-sectional study.

### Inclusion criteria

Consecutive patients with RA, >18 years of age, fulfilling the 1987 American College of Rheumatology (ACR) or the 2010 ACR/European League Against Rheumatism (EULAR) classification for RA, who have attended partial hospitalization program at the department of Rheumatology A, Cochin Hospital, over a 11-month period (May 2016 to April 2017) [12]. All included patients agreed to participate in the study after informed consent, which was recorded in the medical source file. The protocol and the informed consent document have received Institutional Review Board/Independent Ethics Committee (IRB/IEC) approval before initiation of the study (“Comité de Protection des Personnes” Paris Ile de France I).

### Data collection from RA patients

History-taking, physical examination, laboratory tests, and review of medical files were systematically performed to collect data from RA patients. Current and past medication use was obtained from information provided by patients, and based on the review of medical records. RA disease activity was assessed using the Disease Activity Score based on evaluation of 28 joints (DAS28) [13], using Erythrocyte sedimentation rate (ESR) and C-reactive protein (CRP) [14]. Surrogate measures of cumulative disease activity were health status and joint destruction. Health status was measured by the self-administered Stanford Health Assessment Questionnaire (HAQ). Systematic hand and foot x-rays were performed to measure joint destruction, defined by the presence of erosions.

### Laboratory tests

Laboratory studies were obtained in RA patients on the morning of hospital visit. They included complete blood cell count, Westergren erythrocyte sedimentation rate (ESR, considered elevated above 28mm hour-1), CRP concentration (considered elevated if above 10mg/l), and serum creatinine concentration. Rheumatoid factor (RF) and second-generation anti-cyclic citrullinated peptide (anti-CCP2) antibodies were detected by enzyme-linked immunosorbent assay (ELISA).

### Ultrasonography (US) assessment

All of the power Doppler and greyscale ultrasound (PDUS) examinations were performed using a multiplanar technique in accordance with the EULAR guidelines for musculoskeletal ultrasound in rheumatology. PDUS was performed, the day blood samples were collected, by two rheumatologists trained in musculoskeletal US who were blinded to clinical evaluations (APT and ME). Consensus between rheumatologists was obtained before the beginning of the study on both the technique and the US findings, reported in a standardized form. The equipment was a 7–15 MHz linear array transducer (Toshiba Aplio). Power Doppler settings were standardized with a pulse repetition frequency of 750Hz, a gain of 50–53 dB and a low wall filter. US examination was performed on 32 (16 paired) joints of both hands (MCPs 1–5 and PIPs 1–5), both wrists (radio-ulnar, medio-carpal and radio-carpal) and both forefeet (MTPs 1–5).

The presence of hypoechoic synovial hyperplasia (SH) and joint effusion (JE), both assessed using greyscale, and of synovial vascularization, assessed using power Doppler (PD), was scored using semiquantitative scales. The presence of synovitis (SH and PD, without JE) was scored for each joint according to the semiquantitative OMERACT-EULAR-US composite PDUS scale, giving a score of 0–3 for each joint (0 = absence, no synovial hyperemia, 1 = mild, hyperemia in less than 1/3 of the synovial surface area; 2 = moderate, hyperemia in less than 2/3 of the synovial surface area; 3 = marked, in more than 2/3 of the synovial surface area). A global synovitis score, derived from the Global OMERACT-EULAR Synovitis Score (GOESS), was calculated for the 16 paired joints, using the sum of the composite PDUS scores for all joints examined, giving a potential score of 0–96 for the 16 paired joints [15].

### Angiogenic marker measurement

Peripheral blood was collected in a vacutainer tube in the morning, at the same time as samples collected from hospitalized patients for routine analysis. Peripheral blood was allowed to clot by leaving it undisturbed at room temperature. Serum was then prepared by centrifuging whole blood at 1,000–2,000 x g for 10 minutes in a refrigerated centrifuge. Serum concentrations of the following eight angiogenic markers—Vascular Endothelial Growth Factor (VEGF), Placenta Growth Factor (PlGF), Tie-2, Angiopoietin-1, soluble Vascular Cell Adhesion Molecule-1 (sVCAM-1), Interleukin-8 (IL-8, CXCL8), CYR61 (CCN1) and Angiostatin—were measured by quantitative ELISAs (R&D Systems, Minneapolis, MN and RayBiotech, Norcross, GA), according to manufacturer recommendations. Biological function, intra-assay /inter-assay coefficients of variation, recovery and linearity are provided for each marker in **S1 Table**.

### Statistical analyses

All data are expressed as mean values ± standard deviation (SD), unless stated otherwise. Statistical analysis was performed using GraphPad Prism 7.0a software (San Diego, CA). For a two-group comparison, unpaired or paired t-test was used. One-way analysis of variance followed by Tuckey’s multiple comparison tests was performed to compare data among three or more independent groups. Correlations were assessed using Spearman’s rank correlation test. Differences in frequency were examined using the chi-square test. *P < 0.05, **P < 0.01, ***P < 0.001, and ****P < 0.0001.

## Results

### Study population

From the 140 consecutive RA patients initially selected, 15 patients were excluded because of incomplete assessment (absence or incomplete PDUS assessment and/or absence of angiogenic marker measurements). A total of 125 patients (106 females, 84.8%) were finally included, with a mean age of 58.7±15.9 years and a mean disease duration of 15.2±11.5 years. Although the mean DAS-28 was 3.31±1.59, in favor of low disease activity, half of RA patients had a DAS28 >3.2. The majority of patients received conventional synthetic DMARDs (116 patients, 92.8%), corticosteroids (87 patients, 69.6%), and targeted biologic therapies (81 patients, 64.8%), reflecting tertiary center recruitment. Detailed characteristics are provided in **Table 1**.

**Table 1:** Study population.

		Patients with rheumatoid arthritis (n = 125)
**Demographics**	Age (years), mean ± SD Females, n (%)	58.7±15.9 106 (84.8)
**Disease characteristics**	Disease duration (years), mean ± SD Positive rheumatoid factor, n (%) Positive anti-CCP2 antibodies, n (%) Erosions on hand/foot x-rays, n (%)	15.2±11.5 102 (81.6) 107 (85.6) 75 (60.0)
**Disease activity:**	Tender Joint Count, mean±SD Swollen Joint Count, mean ±SD DAS28, mean ±SD DAS28<2.6, n (%) DAS28 >3.2, n (%) DAS28 >5.1, n (%) DAS28 CRP, mean ±SD DAS28 CRP<2.6, n (%) DAS28 CRP>3.2, n (%) DAS28 CRP>5.1, n (%) ESR (mmH1), mean ±SD ESR>28 mmH1, n (%) CRP (mg/L), median (range) CRP >10 mg/L, n (%)	2.7±4.1 3.1±4.5 3.31±1.59 37 (29.6) 62 (49.6) 12 (9.6) 3.02±1.39 51 (40.8) 44 (35.2) 8 (6.4) 18.4±14.7 28 (22.4) 8.7±21.9 25 (20.0)
**Function**	HAQ, median (range) HAQ >1.5, n (%)	0.97±0.82 37 (29.6)
**Ultrasound assessment**	Hand synovitis, n (%) Wrist, n (%) MCP joints, n (%) PIP joints, n (%) Hand tenosynovitis, n (%) MTP joint synovitis, n (%) Positive doppler signal, n (%) + n (%) ++ n (%) +++ n (%)	80 (64.0) 45 (36.0) 63 (50.4) 39 (31.2) 29 (23) 39 (31.2) 53 (42.4) 22 (17.6) 16 (12.8) 15 (12.0)
**Treatment received**	Current corticosteroid use, n (%) Current conventional DMARD use, n (%) Current anti-TNF-α use, n (%) Current rituximab use, n (%) Current tocilizumab use, n (%) Current abatacept use, n (%)	87 (69.6) 116 (92.8) 28 (22.4) 32 (25.6) 12 (9.6) 9 (7.2)

SD: Standard Deviation, DAS: Disease Activity Score, ESR: Erythrocyte Sedimentation Rate, CRP: C-reactive protein, HAQ: Health Assessment Questionnaire, MCP Metacarpophalangeal, PIP: Proximal Interphalangeal, MTP Metatarsophalangeal, DMARD: Disease Modifying Anti-Rheumatic Drug, TNF-α: Tumor Necrosis Factor-α

### Levels of angiogenic markers

Levels of the 8 markers are provided in S2 **Table**. As expected, serum levels of PlGF, a member of the VEGF sub-family correlated with VEGF. Levels of the matricellular protein CYR61 positively correlated with molecules involved in cell adhesion (sVCAM-1, IL-8) or cell death (Angiostatin), and negatively correlated with Angiopoietin-1, implicated in vessel maturation, adhesion, migration, and survival. Levels of soluble Tie-2, a crucial mediator of angiogenic process, correlated with IL-8 and Angiostatin. All correlations between the different markers are presented in S3 **Table**.

### PDUS assessment

Reliability was tested on static images of hand, wrist and forefoot joints obtained from 20 consecutive RA patients. Inter-observer reliability for SH and PD evaluation was defined by κ coefficients of 0.72 and 0.75, respectively. Synovitis was detected in 84 patients with RA (67.2%). Among these patients, 53 patients (42.4%) had positive Doppler signal, including 31 with moderate to marked hyperemia. The global synovitis score ranged from 0 to 52 with a mean value of 5.4±9.9; 29 patients had a global synovitis score >7, corresponding to the 75^th^ percentile value. This cut-off provided the best sensitivity and specificity for active disease, defined by a DAS28 >5.1 (sensitivity: 87.5%, specificity 88%, area under the ROC curve 0.89).

Detailed PDUS evaluation is presented in **Table 1**.

### Levels of circulating angiogenic markers are associated with synovial vascularization assessed by PDUS

#### Angiogenic biomarker levels according to the presence of synovial hyperemia

**Fig 1:**
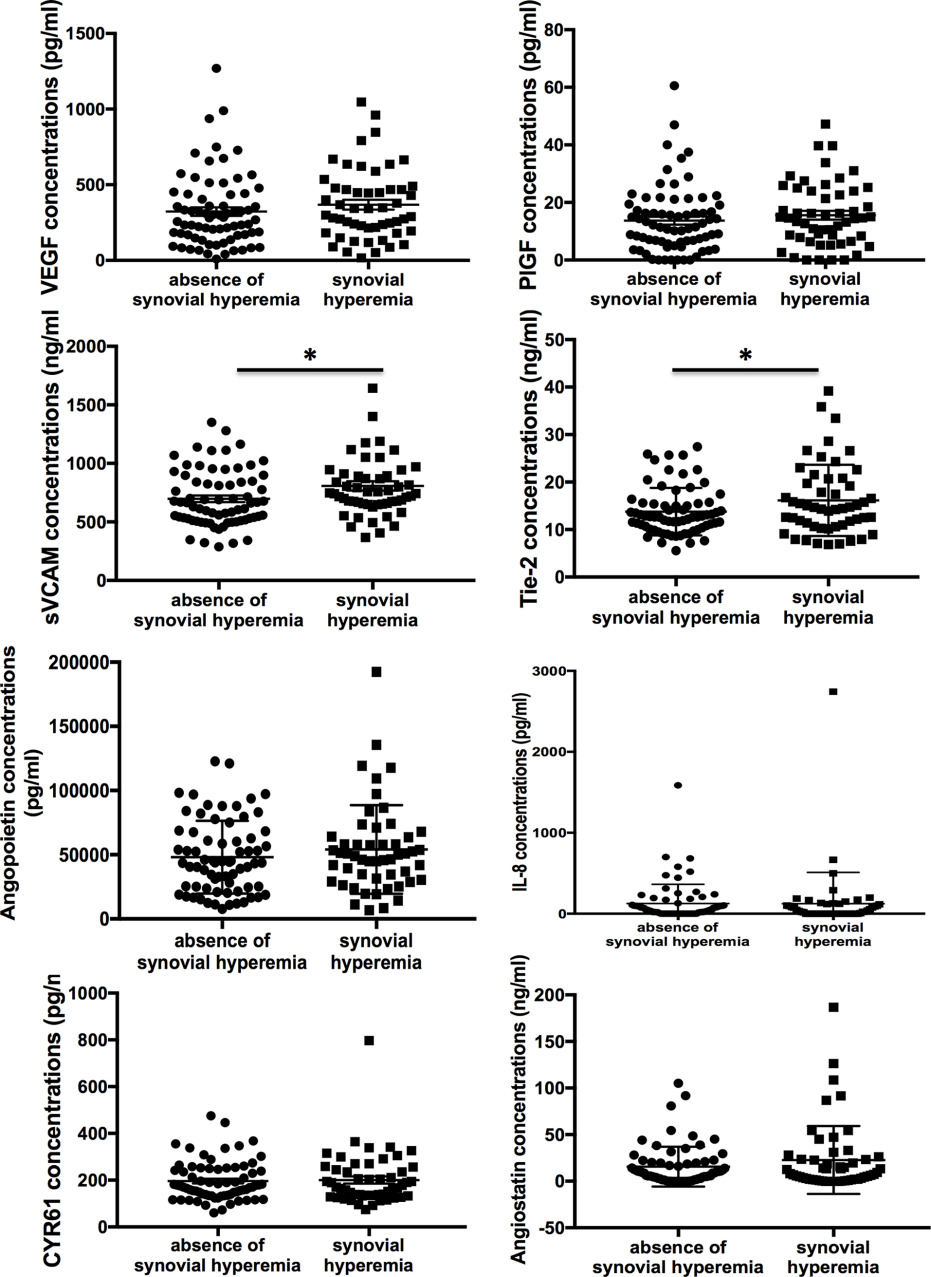
Levels of angiogenic biomarkers between patients without or with synovial hyperemia detected by Power Doppler. Statistical test: two-sided unpaired t-test. * *P*<0.05.

We first compared levels of angiogenic biomarkers between patients without or with increased synovial vascularization detected by PDUS (**Fig 1A–1H**). We observed that serum levels of sVCAM-1 (808±293 ng/mL vs. 697±240 ng/mL, *P* = 0.022) (**Fig 1C**) and Tie-2 (16.2±7.5 ng/mL vs. 13.8±4.9 ng/mL, P = 0.038) (**Fig 1D**) were more likely to be increased in patients with synovial hyperemia detected on at least one joint (Power Doppler grade ≥1).

We next considered relevant synovitis, defined as grade 3 EULAR-OMERACT combined scoring system (Grade 3 Synovial Hypertrophy and ≤ Grade 3 power Doppler signal or Grade 1 or 2 Synovial Hypertrophy and a Grade 3 power Doppler signal) [16]. Forty-nine patients fulfilled this definition. As previously observed, serum levels of sVCAM-1 (816±297 ng/mL vs. 715±260 ng/mL, P = 0.045) and Tie-2 (16.3±7.3 ng/mL vs. 13.9±5.3 ng/mL, P = 0.041) were more likely to be increased in patients with relevant synovitis.

#### Angiogenic biomarker levels according to the extent of synovial vascularization

**Fig 2:**
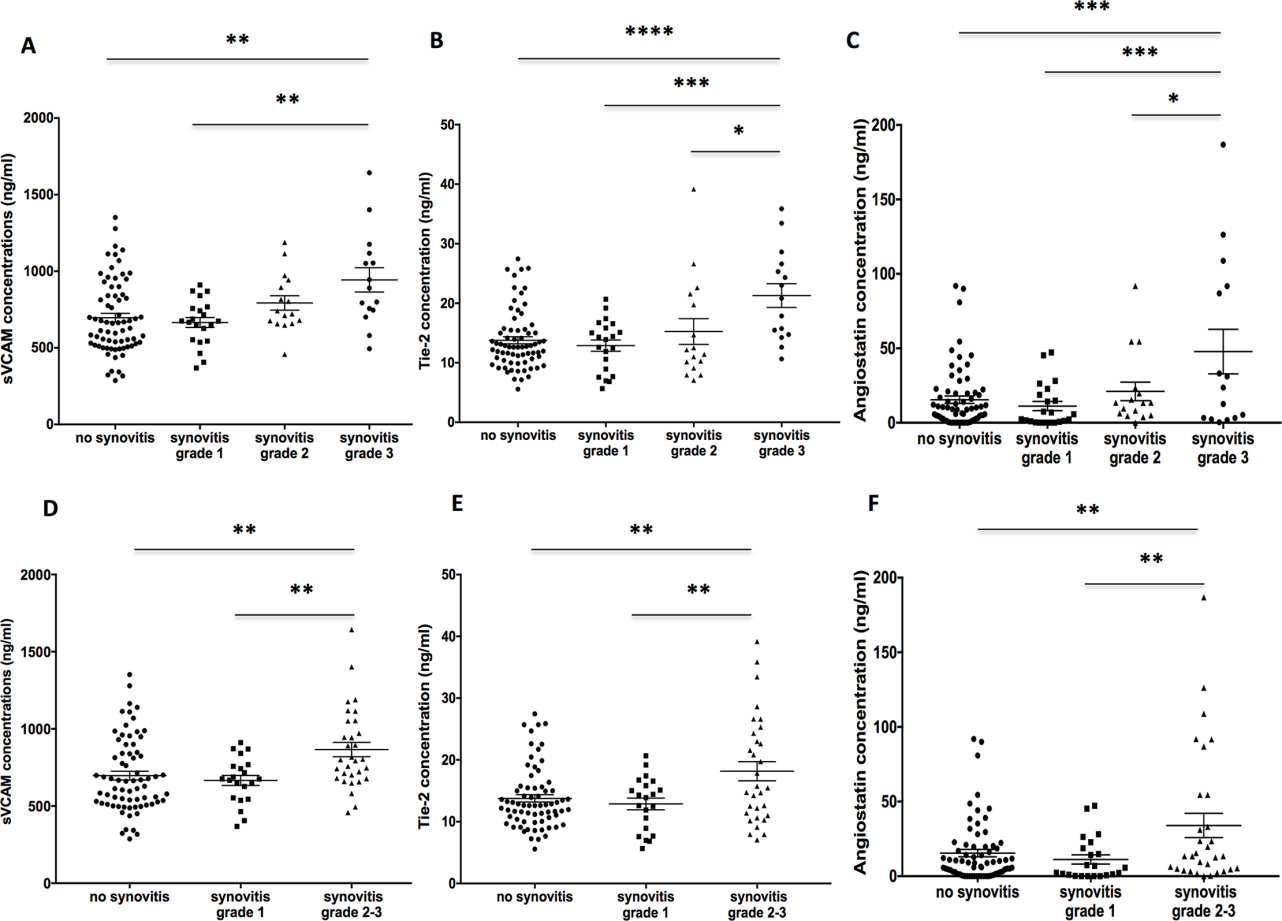
**Levels of sVCAM-1 (A and D), Tie-2 (B and E) and Angiostatin (C and F) according to the intensity and extent of synovial vascularization assessed by Power Doppler Ultrasound**. Statistical test: ANOVA followed by Tuckey’s multiple comparisons test. * *P*<0.05, ** *P*<0.01, *** *P*<0.001, **** *P*<0.0001.

We next stratified these results according the extent and intensity of synovial vascularization assessed by PDUS **(Table 2)**. Interestingly, serum levels of sVCAM-1, Tie-2 and Angiostatin gradually increased together with the grade of the semiquantitative PDUS scale (**Table 2 and Fig 2A–2C**). Indeed, concentrations of these markers were markedly increased in patients with moderate to marked hyperemia (Power Doppler grade 2 and 3) compared to RA patients with absence or mild hyperemia on PDUS (**Fig 2D–2F**).

**Table 2:** Levels of angiogenic markers according to the intensity and extent of synovial hyperemia detected by Power Doppler.

	Absence of synovial hyperemia (n = 72)	Power Doppler Grade 1 (n = 22)	Power Doppler Grade 2 (n = 16)	Power Doppler Grade 3 (n = 15)	P-value
**VEGF (pg/ml), mean (SD)**	309 ± 213	365 ± 222	359 ± 282	382 ± 212	NS
**PlGF (pg/ml), mean (SD)**	13.0 ± 10.2	17.5 ± 11.4	15.0 ± 12.5	13.4 ± 9.0	NS
**sVCAM-1 (ng/ml), mean (SD)**	697 ± 240	666 ± 149	793 ± 189	943 ± 307	* 0.002 ** 0.003
**Tie2 (ng/ml), mean (SD)**	13.8 ± 4.9	12.9 ± 4.3	15.3 ± 8.7	21.3 ± 7.7	* <0.001 ** <0.001 *** 0.025
**Angiopoietin (pg/ml), mean (SD)**	51170 ± 3985	51334 ± 6741	47346 ± 6878	61969 ± 11712	NS
**IL8 (pg/ml), mean (SD)**	105 ± 163	60 ± 85	74 ± 170	93.99	NS
**CYR61 (pg/ml), mean (SD)**	196 ± 85	202 ± 87	218 ± 168	181 ± 59	NS
**Angiostatin (ng/ml), mean (SD)**	15.5 ± 20.4	11.2 ± 14.5	21.1 ± 24.9	47.8 ± 58.1	* <0.001 ** <0.001 *** 0.039

Statistical test: One-way analysis of variance followed by Tuckey’s multiple comparison tests

* Power Doppler grade 3 vs. absence of synovial hyperemia

** Power Doppler grade 3 vs. Power Doppler grade 1

*** Power Doppler grade 3 vs. Power Doppler grade 2

**Fig 3:**
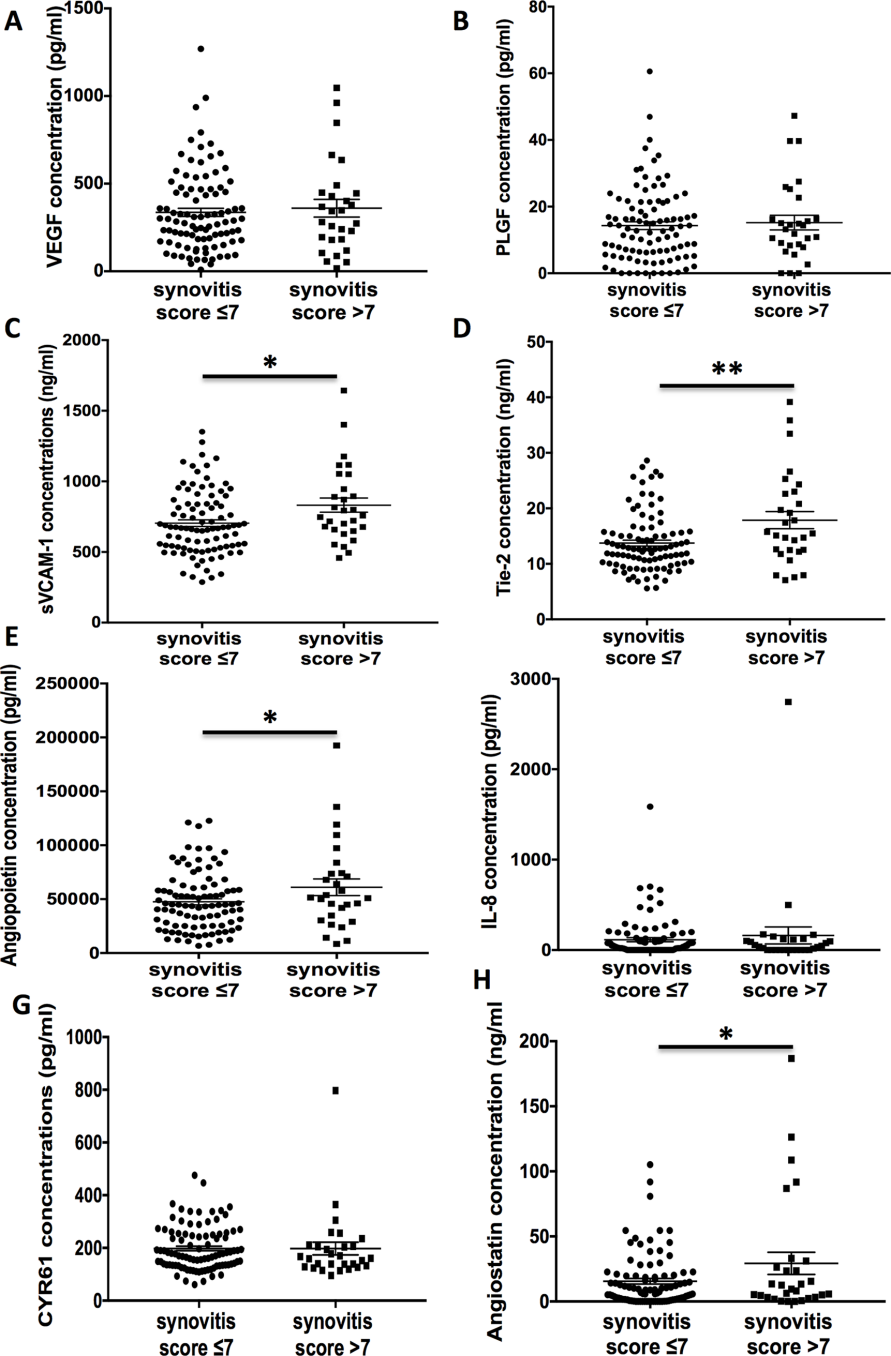
Levels of angiogenic markers according to the values of the global synovitis score. Statistical test: two-sided unpaired t-test. * *P*<0.05, ** *P*<0.01.

Since our previous analysis focused on the highest PDUS semiquantitative scale detected for each patient, we next studied the association between angiogenic marker levels and the global arthritis sum score, to take into account the number of synovitis detected by PDUS and the extent of their vascularization. This global arthritis score correlated with serum levels of sVCAM-1 (r = 0.20, *P* = 0.028), Tie-2 (r = 0.28, *P* = 0.001), and Angiostatin (r = 0.25, *P* = 0.006) (**S1A–S1C Fig**). In addition, patients with a global arthritis score >7 were more likely to have increased sVCAM-1 (832±272 ng/mL vs. 704±227 ng/mL *P* = 0.013), Tie 2 (17.9±8.3 ng/mL vs.13.7±5.2 ng/mL, *P* = 0.002), Angiopoietin-1 levels (61049±40829 pg/mL vs. 47530±27002 pg/mL, *P* = 0.043) and Angiostatin (29.3±45.8 ng/mL vs. 15.5±20.5 ng/mL, *P* = 0.025) than patients with a score ≤7 (**Fig 3A–3H**).

#### Levels of Tie-2 and PlGF are associated with PDUS features indicating residual activity in patients with low disease activity

**Fig 4:**
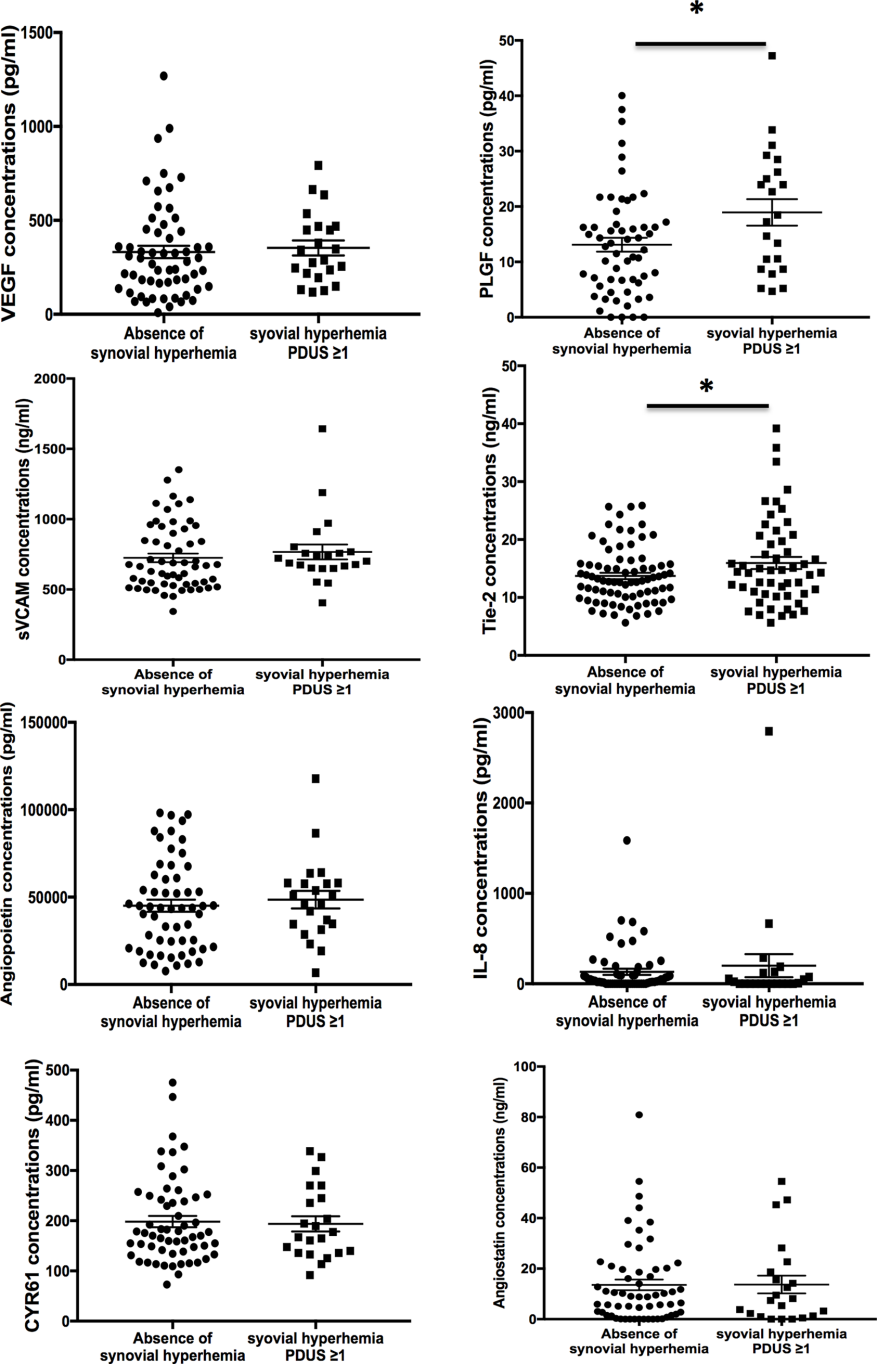
Levels of angiogenic markers according to the intensity and extent of synovial hyperemia detected by Power Doppler in the 81 patients with low disease activity. Statistical test: two-sided unpaired t-test. * *P*<0.05.

We next aimed to determine whether angiogenic markers might be used to detect residual activity in patients with low disease activity and remission. Among the 81 patients with a DAS28 ≤3.2, 22 patients had synovial hyperemia detected on at least one joint (Power Doppler grade 1 in 13 patients, grade 2 in 6 patients and grade 3 in 3 patients). Patients with synovial hyperemia on at least one joint were more likely to have significantly increased levels of PlGF (18.9±11.2 pg/mL vs. 13.1±9.5 pg/mL, *P* = 0.022) (**Fig 4B**) and Tie-2 (15.7±5.8 ng/mL vs. 12.6±3.4 ng/mL, *P* = 0.004) (**Fig 4D**) than patients with absence of synovial hyperemia. No significant difference was observed regarding other angiogenic markers (**Fig 4A, Fig 4C, Fig 4E–4H)**.

Among the 51 patients in remission with a DAS28 <2.6, only 10 patients had synovial hyperemia detected on at least one joint Power Doppler grade 1 in 7 patients, grade 2 in 1 patients and grade 3 in 2 patients). Concentrations of the different angiogenic markers did not significantly differ in patients with or without synovial hyperemia.

#### Angiogenic biomarker levels according to the treatment received

Tie-2 serum levels were significantly decreased in the 32 patients treated with rituximab (12.2±4.6 vs. 17.4±8.2, *P* = 0.003). In line with this observation, a significant reduction of the global arthritis score (1.5±3.2 vs. 7.0±9.9 *P* = 0.004) and of the proportion of patients with a global arthritis score >7 (3/32, 9.4% vs. 14/44, 31.8%, *P* = 0.021) was observed in patients treated with rituximab, compared to the 44 patients treated with conventional synthetic DMARDs only. A trend toward significance was also observed for a lower proportion of patients with moderate to marked hyperemia (Power Doppler grade 2 and 3) upon rituximab (5/32, 15.6% vs. 15/44, 34.1%, *P* = 0.072).

Angiostatin serum levels were significantly decreased in the 28 patients treated with TNF-α inhibitors (8.8±10.3 vs. 26.3±37.6, *P* = 0.045). A marked reduction of the global arthritis score (1.8±3.3 vs. 7.0±9.9 *P* = 0.010) and of the proportion of patients with a global arthritis score >7 (3/28, 10.7% vs. 14/44, 31.8%, *P* = 0.041) was observed in patients treated with TNFα inhibitors, compared to the 44 patients treated with conventional synthetic DMARDs only. A trend toward significance was also observed for a lower proportion of patients with moderate to marked hyperemia (Power Doppler grade 2 and 3) upon TNFα inhibitors (4/28, 14.3% vs. 15/44, 34.1%, *P* = 0.065).

No significant variation was observed regarding the other markers according to treatment received.

#### Angiogenic marker levels according to RA disease characteristics

Patients with DAS28-CRP >5.1 were more likely to have significantly higher Tie-2 (21.8±10.1 vs. 13.9±5.1 ng/mL, *P* = 0.002), CYR61 (288±219 pg/ml vs. 180±177, *P* = 0.026) and Angiostatin (45.8±63.4 vs. 17.5±25.7, *P* = 0.047) serum concentrations, as compared to patients with a DAS28-CRP ≤3.2. No association was observed between other angiogenic markers and disease activity.

VEGF correlated with ESR (r = 0.18, *P* = 0.045) and CRP (r = 0.26, *P* = 0.004) levels. CYR61 correlated with age (r = 0.31, *P*<0.001) and disease duration (r = 0.23, *P* = 0.017). Increased CYR61 levels were observed in patients with bone erosions (218±125 pg/ml vs. 179±69 pg/ml, *P* = 0.041) and HAQ>1.5 (270±170 pg/ml vs. 181±69 pg/ml, *P*<0.001).

## Discussion

This cross-sectional study of 125 RA patients supports the relevance of measuring serum levels of vascular markers to evaluate the intensity and extent of synovial vascularization and, thus, disease activity [17].

Tie-2, a receptor tyrosine kinase expressed primarily in endothelial cells and fundamental for vascular development, was identified as one of the most promising angiogenic marker in our cohort of RA patients, since it increased parallel to the intensity and extent PDUS scale and correlated with the PDUS global arthritis sum score. These findings are consistent with elevated Tie2 expression in human RA synovium [18]. In addition, serum Tie-2 levels were found increased in systemic sclerosis (SSc), another complex disease characterized by widespread microangiopathy [19, 20]. Moreover, a dysregulation of Ang/Tie2 was observed in the bleomycin mouse model, reflecting early and inflammatory stages of SSc, which did not apply for the non-inflammatory tight skin mouse model, highlighting the link between Tie-2 and inflammation, as it was observed in our study [19].

Tie-2 levels markedly decreased together with the PDUS global arthritis score in patients treated with rituximab and were found increased in RA patients in LDA with residual inflammatory activity, together with PlGF levels. In addition, increased PlGF levels were detected in patients with subclinical synovitis. Consistently, higher levels of angiogenic markers, including PlGF, have previously been reported in patients with stringent clinical remission and ultrasound-defined active synovitis [21]. Our findings are consistent with the good correlation previously observed between PDUS and Tie-2 mRNA levels measured in the synovial tissue [22]. These findings also sustain previous data underlining the importance of Tie-2 in the development of synovial neovascularization. Local engagement of synovial Tie-2 signaling is observed during the earliest phases of RA and links the proinflammatory cytokine TNF-α to pathologic angiogenesis [18, 23]. Moreover, gene therapy with soluble Tie-2 receptor inhibits neovascularization and arthritis development and protects against bone destruction in a mouse model of collagen-induced arthritis [24].

VCAM-1 is important in leukocyte trafficking and its increased expression is associated with a number of chronic inflammatory diseases, including RA [25]. Moreover, sVCAM-1 may be important in triggering angiogenesis in the initial stages of RA [26]. sVCAM-1 serum levels gradually increased together with the intensity of synovial inflammation assessed by PDUS. This result is in line with increased sVCAM-1concentrations in RA [25, 27], especially in patients with early and active disease [28, 29], as well as other complex connective tissue disorders like SSc or systemic lupus erythematosus (SLE) [30–32].

sVCAM-1 is a validated biomarker of cardiovascular disease (CVD) and atherogenesis [33], which is of particular importance since CVD has emerged in these recent years as a cause of morbidity and mortality in patients with RA [14, 34]. Our study links for the first time this endothelial activation marker to synovial vascularity assessed by PDUS, highlighting that sVCAM-1 might also be useful as a marker of synovial inflammation that deserves further evaluation in longitudinal studies. This finding is sustained by the arthritogenic role of sVCAM-1 in the development of experimental inflammatory arthritis in the MRL-Fas(lpr) mouse model [35].

Angiostatin has given promising results in cancer therapy trials, as well as preclinical arthritis studies [36]. Serum levels of this anti-angiogenic fragment of plasminogen was found associated with the extent of synovial vascularity in the present study, which is consistent with increased synovial Angiostatin levels observed in patients with inflammatory arthritis [37] and the positive correlation observed in a preliminary study between Angiostatin and cumulative effusion scores evaluated by ultrasonography [37]. Elevated Angiostatin serum levels were also detected in other complex connective tissue diseases like SSc and SLE [38–40].

Being a critical proangiogenic factor, VEGF was expected to be associated with synovial vascularization. However, VEGF levels were not associated with the PDUS semi-quantitative scale and no correlation was observed between VEGF and the global arthritis sum score in the present study. So far, there is no agreement on the association between VEGF and PDUS. Most of previous studies on VEGF had limited sample size and heterogeneous numbers of joints have been evaluated [11, 17, 41–43]. Two prospective studies failed to correlate serum VEGF concentrations with PDUS findings or scores of disease activity [11, 41]. One possible explanation could be variable time response of both factors. Thus, the examination of a high number of joints performed in the herein study is consistent with previous data, suggesting that VEGF is not a powerful marker of active synovitis in RA patients [44, 45].

One novelty of our study was to measure for the first-time serum levels of CYR61, a matricellular protein that is essential for the proper development of the cardiovascular system and the control of angiogenesis. Although we failed to link CYR61 to synovial vascularization in the present study, CYR61 correlated with disease duration and increased CYR61 levels were observed in patients with high disease activity, bone erosions and HAQ>1.5. These findings are consistent with the implication of CYR61 signaling pathway in the development of bone erosion recently described, and the evaluation of CYR61 as a biomarker of structural progression in RA should be further considered [46].

There is no single gold standard for quantifying the level of disease activity in RA. Three composite scores are used for monitoring disease evolution: disease activity score (DAS 28), simple disease activity index (SDAI) and clinical disease activity index (CDAI). The disadvantage of these scores is the degree of subjectivity of some of the criteria. Moreover, a significant proportion of the patients with negative inflammatory tests still have active disease [47], highlighting the need to develop additional tools for a more effective monitoring of the disease. PDUS has demonstrated validity in longitudinal assessment and monitoring of disease activity in RA. It has the ability to detect subclinical synovitis not appreciated by clinical examination alone. This examination also correlates significantly with clinical findings and inflammatory markers, along with synovial histopathology in patients with RA [48]. However, there are certain limitations, including the lack of standardization of PDUS scoring and settings, which can limit the use of this technique in clinical practice. This variability for RA synovitis scores with PDUS [49] may partly explain the lower levels of sVCAM-1 and angiostatin in patients with power Doppler grade 1 compared to patients without power Doppler signals.

Based on our results, angiogenic markers may be used as a surrogate of active synovitis, and their precise place in relation to PDUS deserves further investigation. Angiogenic markers might be more relevant in situations where there are concerns regarding PDUS image acquisition and interpretation, like flash artifacts. The remaining controversies in the number of joints to be assessed for monitoring of disease may also support the first-line use of angiogenic markers to evaluate disease activity. Angiogenic markers might also be considered as an alternative to PDUS in centers where accessibility to this examination is still an issue.

The present study has several strengths. A high number of patients were included and carefully assessed and phenotyped in a tertiary center with a long-lasting experience in RA evaluation and care. Two experienced sonographers performed careful PDUS investigation. The study was performed in a routine clinical setting, which reinforces the external validity of the results. An important joint number was assessed by PDUS, but the addition of even more joints might potentially have modified the findings. Our study is limited by its observational design and any pathogenic link emerged from this type of study should be taken very cautiously. The low number of patients in remission with synovial hyperemia may have prevented the identification of angiogenic markers of subclinical synovitis in patients in remission. Prospective studies will be needed to assess the temporal relationship between disease activity and angiogenic markers.

In conclusion, serum levels of the angiogenic markers Tie-2, sVCAM-1 and Angiostatin were strongly associated with synovial vascularization and inflammation assessed by PDUS among patients with established RA. Moreover, Tie-2 and PlGF were associated with persistent disease activity. These data highlight the possibility to identify surrogate serum angiogenic markers of active synovitis, and their pertinence needs to be confirmed in longitudinal studies.

## Supporting information

S1 Table Biological function, intra-assay and inter-assay coefficients of variation, recovery and linearity of each angiogenic marker. (DOCX)

S2 Table Levels of angiogenic markers in RA patients. (DOCX)

S3 Table Correlation between angiogenic markers in RA patients. (DOCX)

S1 Fig **Correlations between serum levels of sVAM-1 (A), Tie2 (B) and Angiostatin (C) with the global synovitis score**. Statistical test: Spearman’s rank correlation test.
(TIFF)
