# Supplementary material for: Linking systemic angiogenic markers to synovial vascularization in rheumatoid arthritis
Source: PLoS One. 2018 Sep 6;13(9):e0203607. doi: 10.1371/journal.pone.0203607 (PMC6126858; doi:10.1371/journal.pone.0203607)
Supplement: S1 Table — (DOCX) [file pone.0203607.s001.docx]

|  | **Biological function** | **Intra-assay**  **Coefficient of variation** | **Inter-assay**  **Coefficient of variation** | **Average%**  **Recovery** | **Average%**  **Linearity**  **1:2 – 1:4** |
| --- | --- | --- | --- | --- | --- |
| **VEGF** | EC proliferation, migration and survival | 4.5% - 6.7% | 6.2% - 8.8% | 102% | 97% - 97% |
| **PlGF** | EC growth, migration and survival | 3.6% - 7.0% | 10.9% - 11.8% | 96% | 100% - 103% |
| **sVCAM-1** | EC activation and adhesion | 2.3% - 3.6% | 5.5% - 7.8% | No data | 106% - 97% |
| **Angiopoietin-1** | EC migration, sprout formation, and survival | 2.4% - 3.3% | 5.5% - 6.4% | No data | 103% - 93% |
| **Tie-2** |  | 4.3% - 5.3% | 5.2% - 8.5% | No data | 99% - 99% |
| **CYR61** | EC adhesion and migration | 2.0% - 2.3% | 4.9% - 6.4% | 101% | 100% - 107% |
| **Angiostatin** | Inhibition of EC proliferation | No data | No data | 92% | 91% - 93% |
| **IL-8** | EC growth | 5.4%-6.5% | 6.1%-9.7% | 98% | 95% - 94% |

**S1 Table: Biological function, intra-assay and inter-assay coefficients of variation, recovery and linearity of each angiogenic marker**
