# Supplementary material for: Linking systemic angiogenic markers to synovial vascularization in rheumatoid arthritis
Source: PLoS One. 2018 Sep 6;13(9):e0203607. doi: 10.1371/journal.pone.0203607 (PMC6126858; doi:10.1371/journal.pone.0203607)
Supplement: S2 Table — (DOCX) [file pone.0203607.s002.docx]

**S2 Table: Levels of angiogenic markers in RA patients**

| **Angiogenic marker** | **RA patients (n=125)** | |
| --- | --- | --- |
|  | **Mean (SD)** | **Range** |
| **VEGF (pg/ml)**  **PlGF (pg/ml)**  **sVCAM-1 (ng/ml)**  **Tie2 (ng/ml)**  **Angiopoietin-1 (pg/ml)**  **IL8 (pg/ml)**  **CYR61 (pg/ml)**  **Angiostatin (ng/ml)** | 341 (239)  14.5 (11.3)  744 (275)  15.2 (7.2)  52005 (34106)  125 (308)  202 (109)  21.1 (32.4) | 9 - 1269  0.3 - 60.6  287 - 2011  5.5 – 39.3  3755 – 192476  0.1 - 2743  60 - 797  0.1-186.8 |

**SD, Standard Deviation**
