# Supplementary material for: Linking systemic angiogenic markers to synovial vascularization in rheumatoid arthritis
Source: PLoS One. 2018 Sep 6;13(9):e0203607. doi: 10.1371/journal.pone.0203607 (PMC6126858; doi:10.1371/journal.pone.0203607)
Supplement: S3 Table — (DOCX) [file pone.0203607.s003.docx]

**S3 Table: Correlation between angiogenic markers in RA patients**

|  | **VEGF**  **rho**  **(p-value)** | **PlGF**  **rho**  **(p-value)** | **sVCAM-1**  **rho**  **(p-value)** | **Tie2**  **rho**  **(p-value)** | **Angiopoietin**  **rho**  **(p-value)** | **IL8**  **rho**  **(p-value)** | **CYR61**  **rho**  **(p-value)** | **Angiostatin**  **rho**  **(p-value)** |
| --- | --- | --- | --- | --- | --- | --- | --- | --- |
| **VEGF** |  | **0.21**  **(0.039)** | **0.07**  **(0.409)** | **0.12**  **(0.183)** | **0.14**  **(0.127)** | **0.12**  **(0.183)** | **-0.11**  **(0.220)** | **0.13**  **(0.161)** |
| **PlGF** |  |  | **0.06**  **(0.496)** | **-0.05**  **(0.545)** | **0.13**  **(0.154)** | **-0.17**  **(0.052)** | **-0.04**  **(0.686)** | **0.08**  **(0.370)** |
| **sVCAM-1** |  |  |  | **0.06**  **(0.501)** | **-0.09**  **(0.272)** | **0.12**  **(0.202)** | **0.27**  **(0.035)** | **-0.10**  **0.269** |
| **Tie2** |  |  |  |  | **-0.02**  **(0.798)** | **0.25**  **(0.005)** | **0.02**  **(0.770)** | **0.23**  **(0.009)** |
| **Angiopoietin** |  |  |  |  |  | **-0.34**  **(<0.001)** | **-0.38**  **(p<0.001)** | **-0.04**  **(0.644)** |
| **IL8** |  |  |  |  |  |  | **0.19**  **(0.033)** | **0.18**  **(0.047)** |
| **CYR61** |  |  |  |  |  |  |  | **0.18**  **(0.042)** |

**Statistical test: Spearman’s rank correlation test**
