# Supplementary figures and images for: Linking systemic angiogenic markers to synovial vascularization in rheumatoid arthritis
Source: PLoS One. 2018 Sep 6;13(9):e0203607. doi: 10.1371/journal.pone.0203607 (PMC6126858; doi:10.1371/journal.pone.0203607)

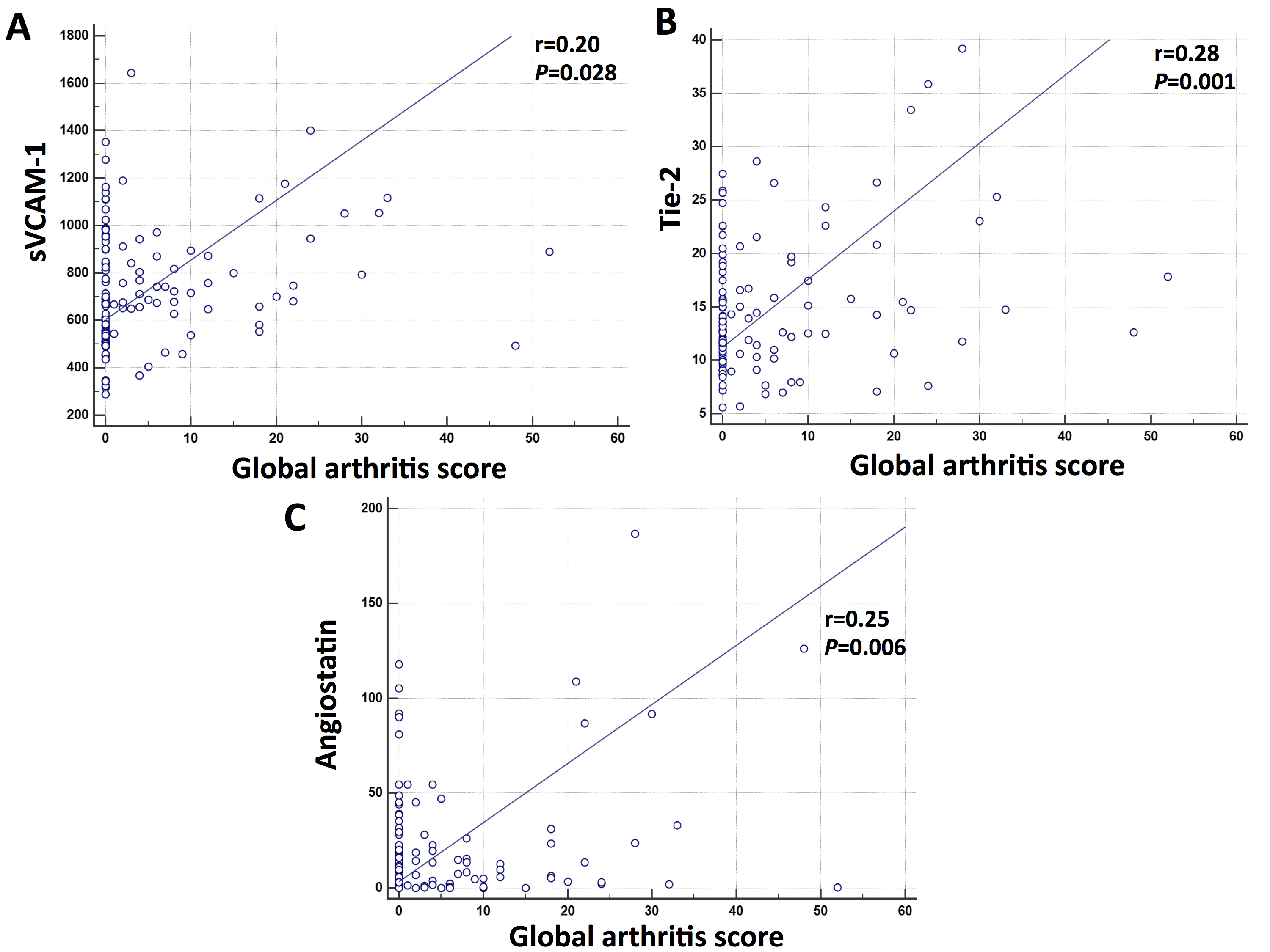

Supplement: S1 Fig — Correlations between serum levels of sVAM-1 (A), Tie2 (B) and Angiostatin (C) with the global synovitis score. Statistical test: Spearman’s rank correlation test. (TIFF) [file pone.0203607.s004.tiff]
